# The logic of conventional and reversed Bateman gradients

**DOI:** 10.1098/rspb.2024.2126

**Published:** 2024-11-06

**Authors:** Jussi Lehtonen, Geoff A. Parker, Camilla M. Whittington

**Affiliations:** ^1^ Department of Biological and Environmental Science, University of Jyvaskyla, Jyväskylä, Finland; ^2^ Department of Evolution, Ecology and Behaviour, University of Liverpool, Liverpool, UK; ^3^ School of Life and Environmental Sciences, University of Sydney, Sydney, New South Wales 2006, Australia

**Keywords:** Bateman gradients, sexual selection, sex roles, reversed Bateman gradients, reversed sex roles

## Abstract

The Bateman gradient is a central concept in sexual selection theory that relates reproductive success to mate number, with important consequences for sex-specific selection. The conventional expectation is that Bateman gradients are steeper in males than females, implying that males benefit more from multiple mating than females do. This claim is supported by much empirical evidence as well as mathematical modelling. However, under some reproductive systems, reversed Bateman gradients are observed, perhaps most notably in syngnathid fishes with male pregnancy. Unlike conventional Bateman gradients, the causal basis of such reversed Bateman gradients has never been modelled mathematically. Here, we present a sex-neutral mathematical model demonstrating how restrictions in capacity for carrying or incubating gametes and embryos (brooding) interact with anisogamy, generating both conventional and reversed Bateman gradients from a single mathematical model. The results clearly demonstrate how anisogamy tends to cause conventional Bateman gradients, but diminishing male brooding capacity under male pregnancy or nesting causes a gradual reversal from conventional to fully ‘reversed’ Bateman gradients.

## 1. Introduction

Reproductive biology shows great diversity in nature, but one repeatedly observed pattern is that males tend to benefit more from mating multiply than females do. The relationship of reproductive success against mate number (the Bateman function [1,2]) typically differs between the sexes: its gradient, the Bateman gradient [2], is steeper in males than in females [3] across most species (see [2,4] for the distinction and link between the Bateman function and the Bateman gradient). Bateman gradients play multiple roles in sexual selection. While they are empirically measurable summary statistics of the intensity of mating competition (the sex with the steeper gradient is expected to compete more strongly for matings), they also offer a partial causal explanation for sexual selection [2,5,6] (see §4 for further detail and some caveats). It is the latter role where mathematical models are particularly important. Furthermore, mathematical models of Bateman gradients can subsequently be used as components of more detailed models of sexual selection or of phenomena such as the evolution of male pregnancy.

**Figure 1.**
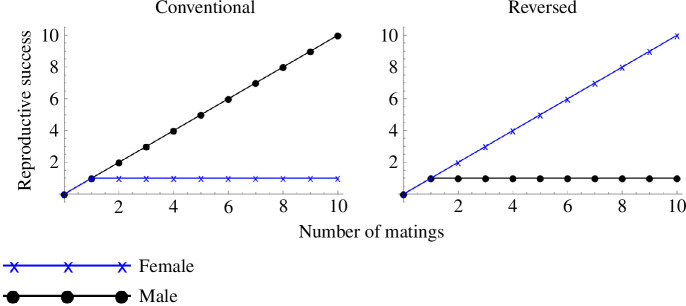
A sketch of conventional and reversed Bateman gradients. The figure presents an extreme example of both cases, where reproductive success for one sex plateaus after one mating, while that for the other increases linearly with the number of matings.

**Figure 2.**
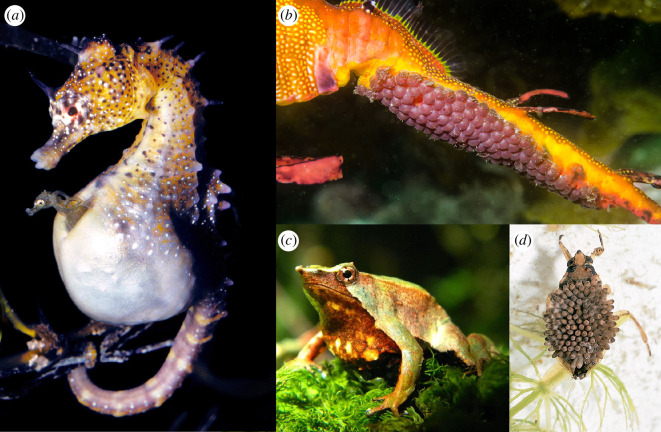
Space for embryo incubation is limited in male brooding species, as in female pregnancy. (*a*) Male *Hippocampus* spp. seahorses carry developing offspring inside an enclosed brood pouch (photo credit: Rudy Kuiter). (*b*) Male *Phyllopteryx taeniolatus* seadragons incubate embryos on an open brood ‘patch’ (photo credit: Tom Burd). (*c*) Male *Rhinoderma darwinii* (Darwin’s frog) brood embryos in the vocal sac (photo credit: Claudio Azat). (*d*) Giant waterbug (*Belostoma flumineum*) males carry developing offspring on their back (photo credit: Kansas Department of Agriculture, Bugwood.org).

Although male Bateman gradients are typically steeper than those for females, there are exceptions where Bateman gradients are reversed, such that females benefit more from multiple matings than males do (figure 1). This phenomenon is best studied empirically in a few fishes: particularly the male-pregnant syngnathids (seahorses, pipefish and seadragons), in which reversed Bateman gradients have been observed [7,8]. Male pregnancy, defined as the brooding of embryos on or in the body of a male (reviewed in [9]), is a rare trait also exhibited by an anuran (*Rhinoderma darwinii*, in which males incubate embryos in a vocal sac) [10], various invertebrates (e.g. the back-brooding waterbug *Belostoma flumineum* (e.g. [11], brooding pycnogonid sea spiders [12) and several other teleosts (e.g. forehead brooding *Kurtus gulliveri* [13], ‘armpit’ brooding dactyloscopids [14], the frogfish skinbrooder *Antennarius caudimaculatus* [15) (figure 2).

While empirical work on Bateman gradients in male-pregnant species is rare, in terms of conceptual and theoretical work, Arnold [16] discussed various combinations of female–male Bateman gradients (including ‘reversed’ ones) but did not explicitly link these to anisogamy or provide a mathematical model. Avise & Liu [17] presented verbal and graphical conjectures on Bateman gradients under male pregnancy and other reproductive systems with a more explicit link to anisogamy (i.e. gamete dimorphism, where, in a species, smaller gametes are produced by males and larger gametes by females), but again did not present a mathematical model. To our knowledge, no formal mathematical model exists linking anisogamy to Bateman gradients under such unconventional reproductive systems. Current models show that the characteristic shape of conventional Bateman gradients is inherent in the mathematics of fertilization ([4]; see also [6]) and provide insight into the causes of conventional Bateman gradients, but these models apply only to systems with either female pregnancy or no pregnancy. Furthermore, while the external fertilizer models of Lehtonen [4] are sex-neutral (the same equation applies to both sexes), the internal fertilization model (which is empirically the most commonly studied case) in the same study is not. Given that a logical and mathematical link from anisogamy to conventional Bateman gradients has been shown, the obvious question becomes: can a similar mathematical framework, generalized so that it applies symmetrically to both sexes, generate both conventional and reversed Bateman gradients depending on the reproductive system? It is important to keep in mind that there are two related but distinct questions: (i) Under what circumstances are Bateman gradients positive in females? and (ii) Under what circumstances are Bateman gradients reversed? In fact, a recent meta-analysis suggests that Bateman gradients are commonly positive in females [18]. The question of reversed Bateman gradients is, however, a much more restrictive one, and a positive female Bateman gradient does not imply that it is steeper than the male Bateman gradient (i.e. reversed). It is also conceptually and theoretically easier to answer question (i) than question (ii): for example, females can potentially benefit from multiple mating whenever the first mating does not guarantee fertilization of all eggs, and subsequent matings improve fertilization prospects.

Here, we develop mathematical models of Bateman gradients accounting for the fundamental constraints that exist in male pregnancy reproductive systems with separate sexes and show that the empirically observed Bateman gradient reversal indeed arises logically from these constraints. The same model accounts for both conventional and reversed Bateman gradients: diminishing brooding capacity causes a gradual reversal from conventional to fully reversed Bateman gradients.

## 2. Methods

Our aim is to construct a model with the following properties: (i) under specific parameter values it should remain compatible with the internal fertilization (i.e. females receive sperm) model of Lehtonen [4] and replicate its results; (ii) it should allow the gamete recipient to be of either sex or mating type (i.e. the model must accommodate male as well as female pregnancy, brooding or nesting); (iii) it should accommodate brood space limitations in the number of gametes the recipient can receive; and (iv) it should accommodate brood space limitations in the number of maturing embryos it can carry (with distinct values for gamete and embryo limits, as in the verbal models of [17]).

We label the gamete recipient with *x* and the donor with *y*—these labels can correspond to either sex, depending on whether we model female or male pregnancy/brooding. Sex is not assigned in the model itself and becomes defined only if parameters are chosen such that the two types produce different numbers of gametes. The number of gametes produced by a single recipient and donor individual is denoted $n_{x}$ and $n_{y}$, and the total number of donor gametes donated to a fertilization arena (i.e. in the female reproductive tract, the male brood pouch, a nest, etc.) is $N_{y}$ (the total number of recipient gametes in the fertilization arena is always simply $N_{x}$ = $n_{x}$, as they all originate from one individual). Following many earlier models (e.g. [4,19–21]), we compute the number of successful fertilizations using a fertilization function first derived by Togashi *et al.* [22] from biophysical principles with no assumptions about differences between the two sexes/mating types (for a review and comparison to other functions, see function $F_{7}$ in table 1 of Lehtonen & Dardare [23]). The fertilization model determines the number of gametic fusions with equal or unequal gamete numbers from each sex/mating type, permitting the sex-neutral labelling of the two types with *x* and *y*. The fertilization function is $f\left(N_{x},N_{y}\right)=N_{x}N_{y}\frac{e^{aN_{x}}-e^{aN_{y}}}{{N_{x}e}^{aN_{x}}-N_{y}e^{aN_{y}}}$ , where *a* is a parameter controlling fertilization efficiency; if $N_{x}=N_{y}$ the function is defined as $f\left(N_{x},N_{y}\right)=\frac{aN_{x}^{2}}{1+aN_{x}}$ [22,23], which can either be derived from the fertilization kinetics on which the model is based, or as the limit of *f* when $N_{y}\to N_{x}$.

Next, we assume a large population with an unbiased sex ratio, and a simple distribution of matings such that in the initial population, all females and males mate exactly *m* times. Following Lehtonen [13], we then consider how a rare ‘mutant’ individual’s (of either sex) fitness depends on its number of matings $\hat{m}$. Using this framework, we can show how variation in the number of matings achieved by a focal individual ($\hat{m}$) influences reproductive success in a given mating environment (with m matings on average) while maintaining an analytically tractable level of complexity in mathematics.

From the recipient perspective (labelled with *x*), the situation is as follows: each recipient produces $n_{x}$ gametes and retains all of them. Each donor mates with *m* recipients, who also mate with *m* donors, and we assume here that each donor divides their gametes evenly over these matings. Therefore, a mutant recipient who mates with $\hat{m}$ donors receives $N_{y}=\hat{m}\frac{n_{y}}{m}$ donor gametes. Here, the first constraint comes into play: the recipient has an upper limit *G* of how many donor gametes it can receive (note that once anisogamy develops, for a female recipient this limit can be very large, and for a male recipient it can be very small). Hence the actual fertilizing set of donor gametes the recipient receives is $\varphi =min\left(N_{y},G\right)=min\left(\hat{m}\frac{n_{y}}{m},G\right).$ Using the fertilization function defined above, the number of fertilizations in this fertilization arena is thus $f\left(n_{x},\varphi \right)$. Now, a second constraint potentially comes into play: the zygotes might grow after fertilization, and it is thus possible that a recipient can retain a smaller number of embryos than it can retain donor gametes (*Z* < *G*, where *Z* is the maximum number of embryos a recipient can carry). A mutant recipient’s reproductive output is thus


(2.1) $$b_{x}\left(\hat{m},m\right)=min(Z,f\left(n_{x},\varphi )\right)$$

where $\varphi =min\left(N_{y},G\right)=min\left(\hat{m}\frac{n_{y}}{m},G\right)$, and (2.1) is the recipient Bateman function.

Now take the donor perspective, which is slightly more complicated. A mutant donor mates with $\hat{m}$ recipients, each of which mate with m-1 additional donors. Therefore, the mutant donor’s mating partners will receive a total of ${N_{y}=n}_{y}/\hat{m}+(m-1)n_{y}/m$donor gametes. Thus, if we assume ‘fair raffle’ competition between donor gametes, the mutant donor gains a fraction $c_{y}=\left(n_{y}/\hat{m}\right)/N_{y}$ of the fertilizations with each recipient. What is the total reproductive success per each of the donor’s mating partners? Again, we must consider recipient gamete retention capacity *G*, so that the fertilizing set of donor gametes his/her partners receive is $\tau =min\left(N_{y},G\right)$ where $N_{y}$ is as above. The number of fertilized gametes per each of his/her partners is then $f\left(n_{x},\tau \right)$ and by the same logic as above, reproductive output per mating partner is $min(Z,f\left(n_{x},\tau )\right)$. Finally, we can write about the mutant donor’s reproductive success:


(2.2) $$b_{y}\left(\hat{m},m\right)=\hat{m}c_{y}\;\;min\left(Z,f\left(n_{x},\tau \right)\right)$$

where all the components of the function are described in the above paragraph. To avoid division by 0, we additionally define $b_{y}\left(0,m\right)=0$ (i.e. with no mating there are no offspring).

We now have compact equations that can describe recipient and donor Bateman functions under e.g. male pregnancy and other reproductive systems where the father can receive a limited number of eggs, or rear a limited number of embryos, or both—but it can equally well describe e.g. female pregnancy where the recipient may be able to receive thousands of donor gametes or more. The model is constructed on a minimal number of fundamental assumptions and restrictions, which are arguably almost necessarily present in nature in some form. While this analytical model considers ‘fair raffle’ situations, we separately investigate the effect of ‘first donor precedence’ using a simulation model the code for which we present in the electronic supplementary material. This is an important addition, because in syngnathids, water bugs and sea spiders that accept eggs from multiple females, we found no evidence in the works cited above in relation to figure 2 that newly deposited eggs can displace older ones or that females or males actively remove eggs from previous females; rather, females simply add eggs to the remaining available male brood space (e.g. water bugs [24]), suggesting first female precedence. Thus, while the analytical model provides a clear exposition of the logic of reversed Bateman gradients under analytically tractable assumptions, it is important to confirm that the results remain qualitatively valid under first female precedence.

## 3. Results

**Figure 3.**
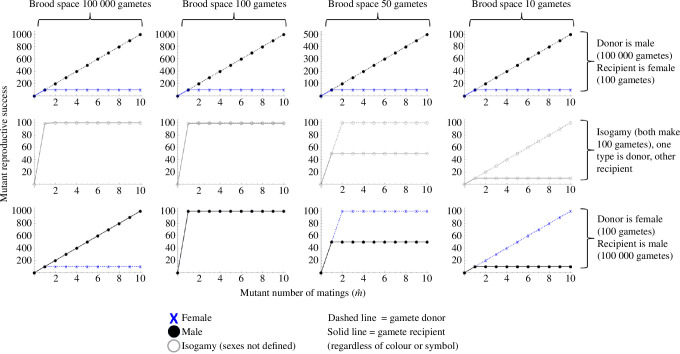
How the gametic system and brooding system influence Bateman gradients. The top left corner illustrates the conventional scenario with female pregnancy. Even when the female’s sperm capacity is hypothetically decreased to unrealistically low numbers (moving to the right along the top row), the conventional Bateman gradient pattern remains. The middle row isolates the effect of brood space limitation by considering the hypothetical combination of isogamy with one mating type being gamete recipient and the other gamete donor: brood space limitation here causes steeper Bateman gradients in the gamete donor. The bottom row considers male pregnancy (and related systems) such that the female acts as gamete donor, and the male brood space varies. While anisogamy maintains conventional Bateman gradients in the bottom left panel, these become entirely reversed as brood space decreases moving to the right along the bottom row. The population is initially monogamous (in all panels, *m* = 1). Fertilization is efficient such that almost all eggs received by a male are fertilized (parameter *a* = 1).

We first zoom out and consider the big picture of the interaction between anisogamy and the constraints arising from the brooding system (figure 3). Here the entire population is initially monogamous (i.e. one mating per reproductive event), and we examine the fitness of a deviant multiply mating individual of both sexes. In our model notation, this implies m=1, while $\hat{m}$ varies on the *x*-axis within each panel in figure 3. Across the panels, we vary two factors: vertically we alter the gametic system, while horizontally we alter gamete storage capacity *G* (which here is assumed to be equal to *Z*). See figure 3 legend for further details.

In figure 3, the top left panel illustrates the conventional situation: females are gamete recipients and can accommodate a very large number of sperm while males are gamete donors, resulting in steeper Bateman gradients for males. The bottom right panel, on the other hand, illustrates the reversed situation where females are gamete donors and males can receive a limited number of eggs, resulting in reversed Bateman gradients. Most of the remaining panels are hypothetical, but by filling in the ‘gaps’ that we do not typically observe in nature, we can expose the logic of both conventional and reversed Bateman gradients. Taken as a whole, figure 3 isolates the effects of three factors on Bateman gradients: anisogamy, brood space limitation and the donor/recipient roles. Let us examine each effect in turn: anisogamy has the overall effect that the type making the smaller gametes (males) tends to have steeper Bateman gradients. This effect appears in the top row, where the male Bateman gradient is steeper regardless of brood space (even for unrealistically small capacity to receive sperm); it also appears in the leftmost panel of the bottom row. The middle row (which represents isogamy), on the other hand, isolates the effect of brood space limitation from the effect of anisogamy: its two rightmost panels show that in the absence of anisogamy but in the presence of limited brood space, Bateman gradients are steeper for the gamete donor. Thus, figure 3 is consistent with both classical predictions (top left) and reversed Bateman gradients (bottom right). For the model to be logically consistent, a fundamental constraint that must be fulfilled is that (given an even sex ratio) the average reproductive success of females must equal that of males; this is often referred to as the ‘Fisher condition’ [25]. In the analytical model, this is easy to confirm: because deviant individuals are vanishingly rare, population mean reproductive success is determined by the ‘resident’ type, and thus the female and male curves must coincide at *m* matings as they do in all figures representing the analytical model.

**Figure 4.**
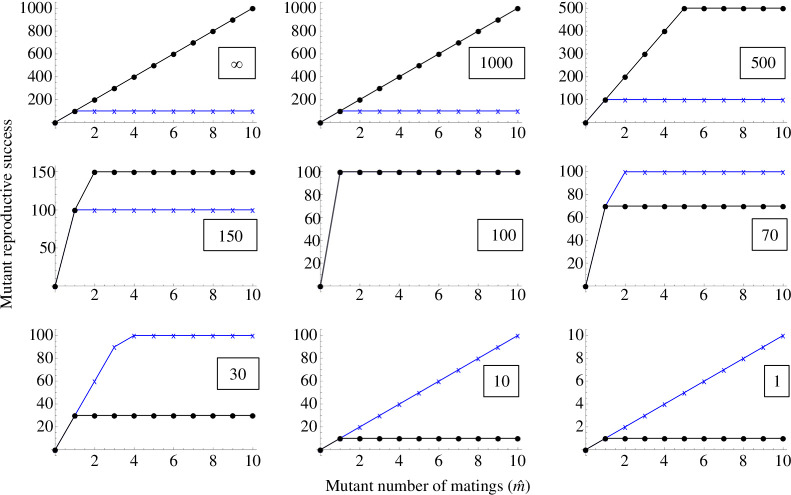
The gradual reversal in Bateman gradients as the size of the male brood space (or more generally, fertilization arena) becomes limiting. Blue crosses and lines represent female reproductive success, as per equation (2.1). Black circles and lines represent male reproductive success, computed with equation (2.2). The number of female gametes (*G*) a male can fit in his brood space is indicated in a box on each panel, and here it is assumed to be the same as or greater than the number of embryos the male can carry (Z). *G* declines from the top left to the bottom right panel. Here it is assumed that the population is initially monogamous (*m* = 1). Fertilization is efficient—almost all eggs received by a male are fertilized (parameter *a* = 1), and *n_x_* = 100 000 and *n_y_* = 100. When brood space is not limiting (top left), we have the conventional male-steeper Bateman gradient. In principle, the gradients can reverse entirely if the brood space is small enough (bottom right). Blue lines and crosses represent females, black lines and dots represent males.

**Figure 5.**
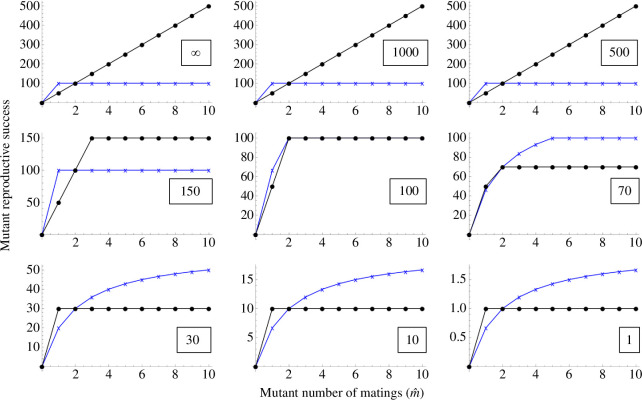
As figure 4, but with a polygamous population (*m* = 2 in all panels). Other parameters are as in figure 4. There can be egg competition for brood space, and polygyny and the resulting egg–egg competition for brood space reduces the difference between female–male Bateman gradients (compare for example the last panels of figures 4 and 5). Again, fertilization is efficient such that almost all eggs received by a male are fertilized. Blue lines and crosses represent females, black lines and dots represent males.

**Figure 6.**
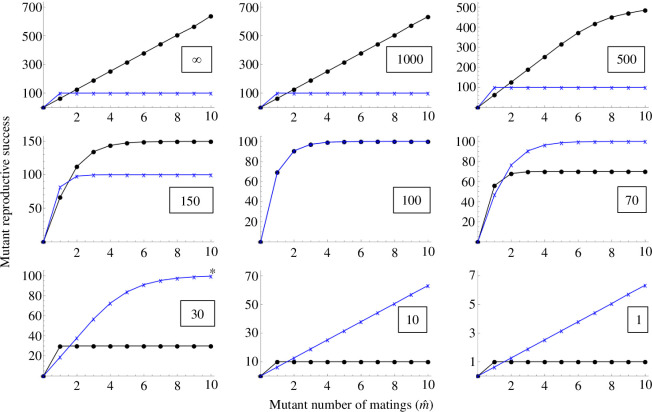
Results of a simulation with first female precedence. While the implementation of the underlying model is very different, the visual logic of the figures is exactly as in figures 4 and 5. Females make 100 eggs, males make a very large number of sperm so that it is not limiting. Blue lines and crosses represent females, black lines and dots represent males.

Having established that the model can explain the broad outlines of Bateman gradients under variation in gametic and reproductive systems, we now focus on how limited male brood space can influence Bateman gradients under typical anisogamy ratios and male pregnancy or related reproductive systems. Again, we initially assume that the number of embryos a male can carry is at least equal to the maximum number of eggs a male could carry (*Z ≥ G* in our model notation). This could correspond to, for example, shrinkage or constant size of the developing embryo, or to a brood pouch that can stretch to accommodate growing embryos so that no eggs need to be discarded. We will later consider a scenario where *Z < G*, but in figures 4–6 we have *Z ≥ G*.

Figures 4 and 5 show the transition of the bottom row of figure 3 in finer detail: increasingly limited brood space under male pregnancy gradually causes the sex-specific Bateman gradients to reach equality and finally reverse entirely. The difference between figures 4 and 5 is that the former presents an initially monogamous population (as in figure 3), while figure 5 shows the same effect in an initially polygamous population (*m* = 2) with the consequence that gamete donors face egg competition from other individuals. While egg competition slightly decreases the difference between sex-specific Bateman gradients (in line with earlier theory [4,26]) particularly in the bottom row, it does not change the qualitative conclusions. The simulation results of figure 6 show that these qualitative results are not altered by first female precedence either. Although not as visually clear from the figure as it is with the analytical model, the ‘Fisher condition’ is also fulfilled in the simulation results. In this case, the consistency arises directly from the simulation structure, where in each mating event, the same fertilized eggs add simultaneously to the reproductive success of one individual of each sex.

**Figure 7.**
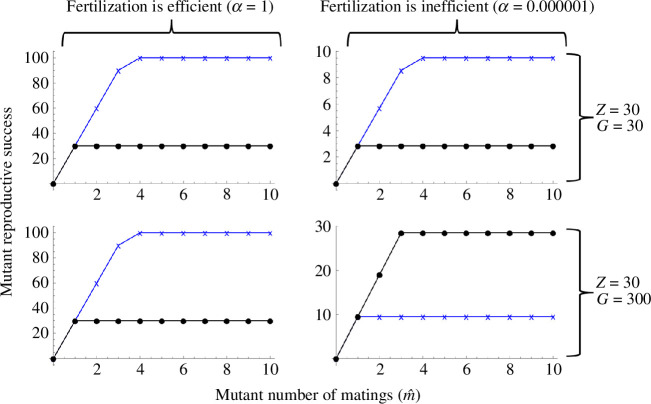
A theoretical ‘reversal of reversal’ effect. In a study by Lehtonen [4], Bateman gradients could be reversed under internal fertilization, if fertilization was very inefficient. Under male pregnancy, extreme conditions (see text) can cause reversed Bateman gradients to reverse again so that they become conventional Bateman gradients. Fertilization is efficient in the two panels on the left (parameter *a* = 1), and very inefficient in the two panels on the right (*a* = 0.000001). In all panels males can carry at most 30 embryos (*Z* = 30). In the top row, males can also only carry 30 eggs (*G* = 30), while in the bottom row they can carry 300 eggs (*G* = 300, whereas they can still only hold 30 embryos which are now considered to be significantly larger than eggs). *m* = 1, *n*_x_ =100,000 and *n*_y_ =100 in all panels. Blue lines and crosses represent females, black lines and dots represent males.

The internal fertilization (with female as recipient) model of Lehtonen [4] of conventional Bateman gradients showed a surprising theoretical result: Bateman gradients can hypothetically be reversed by very inefficient fertilization even when females are gamete recipients and male donors, although this effect seems rather unlikely in nature. Importantly, the effect is completely independent of the ‘male pregnancy’ type of reversal presented above. Therefore, one might think that the two reversal effects could in principle cancel each other out. It turns out such a ‘reversal of reversal’ could hypothetically happen, but under a very specific set of circumstances: fertilization must be very inefficient, and males must be able to receive a large number of eggs relative to the number of embryos they can carry (figure 7).

## 4. Discussion

Since Bateman’s work more than 75 years ago [1], Bateman gradients have become a central topic of study in sexual selection research (e.g. [2,3]), and have also become surprisingly controversial (e.g. [27]). While Bateman gradients do not by themselves directly tell us how sex-specific selection ultimately acts on traits in the two sexes (see e.g [2,5,28,29], for the theoretical and causal role Bateman gradients play), they are nevertheless intimately linked to questions on sexual selection and the evolution of sex-specific traits we tend to associate with females and males (so-called ‘sex roles’: e.g. [30]).

There is both an empirical and a logical side to these debates and to the importance of Bateman gradients. Bateman’s original empirical methodology has been criticized (e.g. [27,31]), but similar experiments have later been conducted numerous times, and the big picture of Bateman’s conclusions generally holds up (e.g. [3,32]). At the same time, we now know that this big picture is more nuanced (e.g. Bateman gradients are quite commonly positive in females: [18]). But aside from the growing empirical understanding, the consistency of the internal logic of theory is equally important, particularly given that theory in sexual selection and sex role evolution has at times been criticized for missing or circular logic (e.g. [33,34]; see also [35] for discussion). While a mathematical and logical basis for conventional Bateman has recently been shown [4], in a fully consistent theory, it is equally important to demonstrate how deviations from the conventional pattern can arise. That is what we have done here: we have shown, from first principles using a mathematical model, how one model with no pre-defined ‘males’ and ‘females’ gives rise to both conventional and reversed Bateman gradients depending only on the parameter combinations. In the conventional scenario where females receive gametes from males and can accept a very large number of sperm, we find conventional Bateman gradients (top left panel of figure 3). In the opposite scenario where males receive gametes from females and can accept a very limited number of eggs, we find reversed Bateman gradients (bottom right panel of figure 3). Thus, both conventional Bateman gradients and reversed Bateman gradients in commonly studied mating systems arise from a single logical and mathematical structure that has no pre-assigned ‘male’ or ‘female’ built into it. Note that the reversed Bateman gradients modelled here are very different from the hypothetical reversed Bateman gradients found in Lehtonen [4], which require an unlikely combination of assumptions; a similar hypothetical effect reappears in figure 7, where in principle the Bateman gradient reversal due to brood space limitation can be secondarily re-reversed—but again, only under an unlikely combination of assumptions. The main results in figure 3–6, however, do not require any unrealistic assumptions.

Thus, anisogamy causes gamete numbers to be male-biased so that ova become a limited resource for males, typically resulting in steeper male Bateman gradients (for theory, see top row of figure 3 in this study and [4]; for evidence, see [3]). On the other hand, our present model shows that when males provide a limiting resource necessary for female reproduction (in our model, brood space for eggs to develop), Bateman gradients can be entirely reversed. Rather than an opposite-sex resource constraint operating on just males (limited female gametes through anisogamy), we now also have an opposite-sex resource constraint acting on females (with the inclusion of limited male brood space). In figures 3–6 we see that under anisogamy and when brood space is not limiting, typical Bateman gradients apply regardless of which sex is in the role of gamete donor and recipient, with the male gradient steeper than the female gradient. Reversed Bateman gradients are generated beyond the central panel in figures 4–6 (i.e. when male brood space becomes limiting). While this ‘dual constraint case’ can cause Bateman gradient reversal, the anisogamy constraint generates wider parameter space for the typical, male-steeper Bateman gradients (figure 3).

Why do our results depend on anisogamy? An earlier model of Bateman gradients under internal fertilization (model 3 in [4]) demonstrated how anisogamy leads to steeper Bateman gradients in males, showing the transition from equality of gradients under isogamy to the typically male-biased gradients under anisogamy. However, model 3 in [4] was ‘asymmetric’ in the sense that the roles of the two sexes as donor and recipient were fixed, as were the equations used to model females and males: the model was silent on male pregnancy and related reproductive systems. Here, we have a single model where either sex can play either role, and it is the parameter values that retrospectively determine the sexes. When females play the role of gamete recipient and can accommodate a large number of sperm, the model coincides with that of Lehtonen [4]. In contrast, when females play the role of donor and males can receive a limited number of eggs, the model corresponds to reproduction via male pregnancy and related systems with brood limitation on the male side. Note that while reversed Bateman gradients occur and are of great interest, they are relatively rare in metazoans. We stress that while we see anisogamy as having a central role in determining an ancestral flow towards male–male competition in sex role evolution [36], its effect appears to saturate rapidly with little or no further correlation to the magnitude of anisogamy (for theoretical reasoning why this is the case, see Janicke *et al.* [3], who found a binary correlation; Mokos *et al.* [37], who did not find a sustained correlation beyond that; and Lehtonen & Parker [38] ). Ecological, sociobiological and other factors both shape the degree of anisogamy and will modify Bateman gradients and can (in certain rather rare cases) secondarily reverse the typical male-steeper Bateman gradient pattern.

This is, to our knowledge, the first mathematical model of Bateman gradients under brooding constraints. It has direct application to species in which male brood space is limited and there is no sperm competition between males (some examples are provided in electronic supplementary material, table S1, indicating which aspects of their biology fit the current model and which do not). In this article, our aim has been to understand some of the systems where reversed Bateman gradients are commonly studied—male pregnancy and some cases of male nesting (termed ‘external male pregnancy’, by [17], defined as males who tend to eggs from one or more females, with little sperm competition). With our approach, we deliberately kept the number of parameters and complexity of the model minimal, showing areas of parameter space where reversed Bateman gradients are theoretically expected and which broadly match with empirical observations in syngnathid fishes (e.g. [39]). It is important to note that we do not suggest that this is the only way reversed Bateman gradients can arise; in the future, we suggest that further models could be constructed for other systems. For example, a few birds (e.g. wattled jacanas, *Jacana jacana*) show sex role reversal and males brood their offspring [40], with possibly analogous results. Such a model would need to account for females initially receiving gametes from multiple males who potentially face sperm competition, and then subsequently donating fertilized eggs to brooding males.

Our work also has implications for the ongoing discussion on the definitions of sexual selection. In his pioneering survey, Darwin [41] defined sexual selection solely in terms of competition for mates. His definition was extended to include competition for access to gametes, which allows inclusion of post-ejaculatory traits [42]. Though it usefully broadened the scope of sexual selection, Janicke [43] has recently pointed to certain flaws in this wider definition, proposing that a better and more general definition should be based on competition for resources provided by the other sex (note that ‘provided by the opposite sex’ is important, since it can be argued that natural selection covers competition for resources in general). Our analysis shows that a limiting resource for females, such as brood space provided by males, can generate higher Bateman gradients in females than males. Thus while anisogamy results in female gametes becoming the limiting resource that commonly generates higher Bateman gradients in males, Janicke’s wider definition seems more appropriate, covering cases of ‘sex role reversal’, where females do not compete for male gametes, but some other limited resource provided by males, such as brood space.

## Data Availability

Supplementary material is available online [44].
